# Copper oxide-based cathode for direct NADPH regeneration

**DOI:** 10.1038/s41598-020-79761-6

**Published:** 2021-01-08

**Authors:** J. T. Kadowaki, T. H. Jones, A. Sengupta, V. Gopalan, V. V. Subramaniam

**Affiliations:** 1 Applied Physics Laboratory, Department of Mechanical and Aerospace Engineering, The Ohio State University, Columbus, USA; 2 Department of Chemistry and Biochemistry, The Ohio State University, Columbus, USA; 3 Department of Chemistry and Biochemistry, Center for RNA Biology, The Ohio State University, Columbus, USA

**Keywords:** Biochemistry, Biotechnology, Energy science and technology, Engineering, Materials science, Nanoscience and technology

## Abstract

Nearly a fourth of all enzymatic activities is attributable to oxidoreductases, and the redox reactions supported by this vast catalytic repertoire sustain cellular metabolism. In many biological processes, reduction depends on hydride transfer from either reduced nicotinamide adenine dinucleotide (NADH) or its phosphorylated derivative (NADPH). Despite longstanding efforts to regenerate NADPH by various methods and harness it to support chemoenzymatic synthesis strategies, the lack of product purity has been a major deterrent. Here, we demonstrate that a nanostructured heterolayer Ni–Cu_2_O–Cu cathode formed by a photoelectrochemical process has unexpected efficiency in direct electrochemical regeneration of NADPH from NADP^+^. Remarkably, two-thirds of NADP^+^ was converted to NADPH with no measurable production of the inactive (NADP)_2_ dimer and at the lowest reported overpotential [− 0.75 V versus Ag/AgCl (3 M NaCl) reference]. Sputtering of nickel on the copper-oxide electrode nucleated an unexpected surface morphology that was critical for high product selectivity. Our results should motivate design of integrated electrolyzer platforms that deploy this heterogeneous catalyst for direct electrochemical regeneration of NADH/NADPH, which is central to design of next-generation biofuel fermentation strategies, biological solar converters, energy-storage devices, and artificial photosynthesis.

## Introduction

Electron transfer is key to all cellular metabolism as redox reactions undergird the work performed by all living organisms^1,2^. During the exergonic oxidation of nutrients and foods, catabolic processes first capture electrons in the form of coenzymes (e.g., reduced form of nicotinamide adenine dinucleotide, NADH) and then generate cellular energy currencies by harnessing the electromotive force created during electron transport from these reduced coenzymes to oxygen. In contrast, specialized coenzymes (e.g., reduced form of nicotinamide adenine dinucleotide phosphate, NADPH) are used to support reductive syntheses during anabolism. Not surprisingly, metabolism is often a target of cancer therapies^3^.

Reduced coenzymes also have potential applications in bioinorganic artificial photosynthesis^4,5^, and are vital for biocatalyst-centered synthetic efforts in the pharmaceutical and (bio)chemical industries^6–11^. However, economic viability of these approaches depends on lowering the cost of reduced coenzymes. For example, inexpensive production of butanol from lignocellulosic biomass via fermentation would provide an economically appealing additive to gasoline were it not for the production cost of butanol^12^. We address this crippling roadblock by exploiting a nanostructured Ni–Cu_2_O–Cu heterolayer material for photoelectrochemical regeneration of NADPH.

There have been enzymatic, chemical, electrochemical, and biological approaches to regenerate NADH, but each has drawbacks in terms of cost, yield, ease of use, scalability, and efficacy for a variety of reasons^6,10,11^. Among these methods, electrochemical regeneration is the most direct, requiring few to no intermediate steps^6,10^. However, challenges in the artificial regeneration of NADH (and by extension, NADPH) include: (i) the formation of inactive forms, particularly the (NADP)_2_ dimer and, to a lesser extent, the inactive isomer 1,6-NAD(P)H, both of which affect the purity and utility of the product (Supplementary Fig. S1); (ii) the need for high overpotentials which can also lead to unwanted products, corrosion, and degradation of electrodes; and (iii) the requirement of expensive catalysts such as Rh, Ru, Ir, and Pt^6,10,13^. Bare metallic materials favor production of the inactive (NADP)_2_ dimer during direct electrochemical regeneration of NAD(P)H and precious metals such as Pt, Ir, and Ru can increase selectivity toward 1,4 NAD(P)H^13^. NADH has also been regenerated using photocatalytic and photoelectrocatalytic approaches^14,15^. Recently, a photoelectrochemical process requiring collimated focused 625-nm radiation and expensive electrode materials (platinum and p-type GaAs), was used to regenerate NADH^15^; without the platinization of the p-type GaAs surface, only the inactive dimer (NAD)_2_ was produced^15^.

Here, we show that: (i) a nanostructured, heterolayer electrode made from widely available and inexpensive materials (Ni, Cu) and a novel photoelectrochemical surface-modification process can be used to electrochemically regenerate active NADPH from NADP^+^ with a mild overpotential (− 0.75 V versus Ag/AgCl (3 M NaCl) reference), (ii) reduced cofactors can be produced directly without the need for an expensive catalyst, and (iii) the regenerated NADPH is notably devoid of the inactive dimer that is typically generated in similar undertakings. The activity of the regenerated NADPH was evaluated using an alcohol dehydrogenase assay, a choice that was inspired by our desire to demonstrate the utility of this method for generation of biofuels from biomass. The latter assay, together with mass spectrometry or NMR data, is important in confirming the *bona fides* of the end product; to establish the purity, we sought to not rely solely on electrochemical voltammetry and UV–vis absorption measurements since reaction products may contain the dimer in addition to inactive isomer^16,17^. Finally, unlike previous attempts that demonstrated photoelectrochemical regeneration of NADH^15^, we provide evidence here that illumination and electrochemistry need not be concurrent, implying that direct electrochemical regeneration can proceed without the undesirable production of the inactive dimer. Our facile, cheap, and direct electrochemical regeneration of NADPH, especially without any inactive dimer, is a useful advance in the regeneration of cofactors^18,19^.

## Results

### Ni–Cu_2_O–Cu cathode is a nanostructured heterolayer

CuO and Cu_2_O are both p-type semiconductors (1.3–1.7 eV and 2.0–2.5 eV, respectively)^20,21^. The photo-galvanic properties of the interfaces between semiconductors and electrolytes have been extensively studied since Edmond Becquerel’s first report in 1839^22–24^. Copper oxide-based electrodes have since been used for hydrogen production^20,25,26^, photoelectrocatalytic conversion of CO to liquid fuels (ethanol, acetate, and n-propanol)^25^, and solar energy conversion^26–30^. The Becquerel effect has been used in photoelectrocatalytic regeneration of the cofactor NADH, but with exotic and expensive materials (Pt-modified p-GaAs semiconductor electrodes) and requiring the presence of both illumination and applied bias^15^. Here, we use photoelectrochemical surface modification of the Ni-Cu_2_O-Cu electrode to directly electrochemically regenerate NADPH from NADP^+^ using inexpensive materials (Ni and Cu) and, importantly, without the need for the simultaneous presence of illumination. The efficacy of the process, was evaluated based on the activity of the NADPH product, which in turn was assessed using an enzymatic assay based on *Lactobacillus brevis* alcohol dehydrogenase (*Lb*ADH)^31^.

We prepared copper oxide-based cathodes by a single-step electrochemical process on copper 100 mesh substrates (Alfa Aesar 45,186, woven from 0.11 mm dia. Wire, 1 cm^2^). Copper mesh was selected as a working electrode because it is inexpensive, and it provides a higher surface area-to-volume ratio compared to planar foils. Prior to electrodeposition, the meshes were cleaned by two 10-min sequential sets of sonication in absolute ethanol and de-ionized water for another 10 min. The meshes were dump rinsed in de-ionized water between each sonication step. Finally, the meshes were dried with clean, compressed air. The copper oxide layer was electrodeposited using a solution containing 0.48 M CuSO_4_, 3 M lactic acid. This solution was prepared using copper sulfate pentahydrate (Sigma-209198), concentrated lactic acid (Ricca RABL0010-500A) and deionized water (18.2 MΩ-cm, Milli-Q)^32^. The pH of the solution was then adjusted to 11 by adding pure NaOH. The electrodeposition process was carried out potentiostatically (Gamry Interface 1000) at − 0.5 V vs Ag/AgCl (3 M NaCl, Basi MF-2052) for 2 h at room temperature (20–25 °C) in a two-compartment cell (Supplementary Fig. S2). The cell was built using an agarose bridge (2% agar, Invitrogen 15110-019) and a coiled (~ 3 turns, 0.404 mm diameter) Pt-wire (Alfa Aesar 45,058) counter electrode. The cathode chamber was filled with cupric lactate solution and the anode chamber was filled with a 0.5 M potassium phosphate (pH 7) solution to prevent both degradation of the Pt wire and adsorption of Cu. Details of the copper oxide electrodeposition reaction are provided in the supplement (Supplementary Eq. S1).

The electrodeposited material was reddish in appearance, a characteristic typical of Cu_2_O. This nanostructured heterolayer cathode was then further modified by DC sputter deposition (EBTEC Co.). A thin nickel coating was deposited onto the Cu_2_O–Cu heterostructure by sputtering a Ni target (5.1 cm diameter, polished with 100 grit sandpaper before deposition) in Ar gas (~ 600 mTorr) at 340 V, 12.5 mA and at a working distance of 3 cm between the sample and Ni target. Since we sought to reduce NADP^+^ to NADPH, Ni was selected because of its well documented ability to effectively adsorb hydrogen^33^. The sputtering process was carried out for 96 h after which the sample was overturned, and the sputtering process repeated for 24 h. Wires (22 AWG tinned Cu) were then attached to the Ni–Cu_2_O–Cu electrode with conductive silver epoxy (MG Chemicals 8331) to decrease any uncompensated contact resistance (i.e., IR drops) in subsequent measurements of the electrode potential.

**Figure 1:**
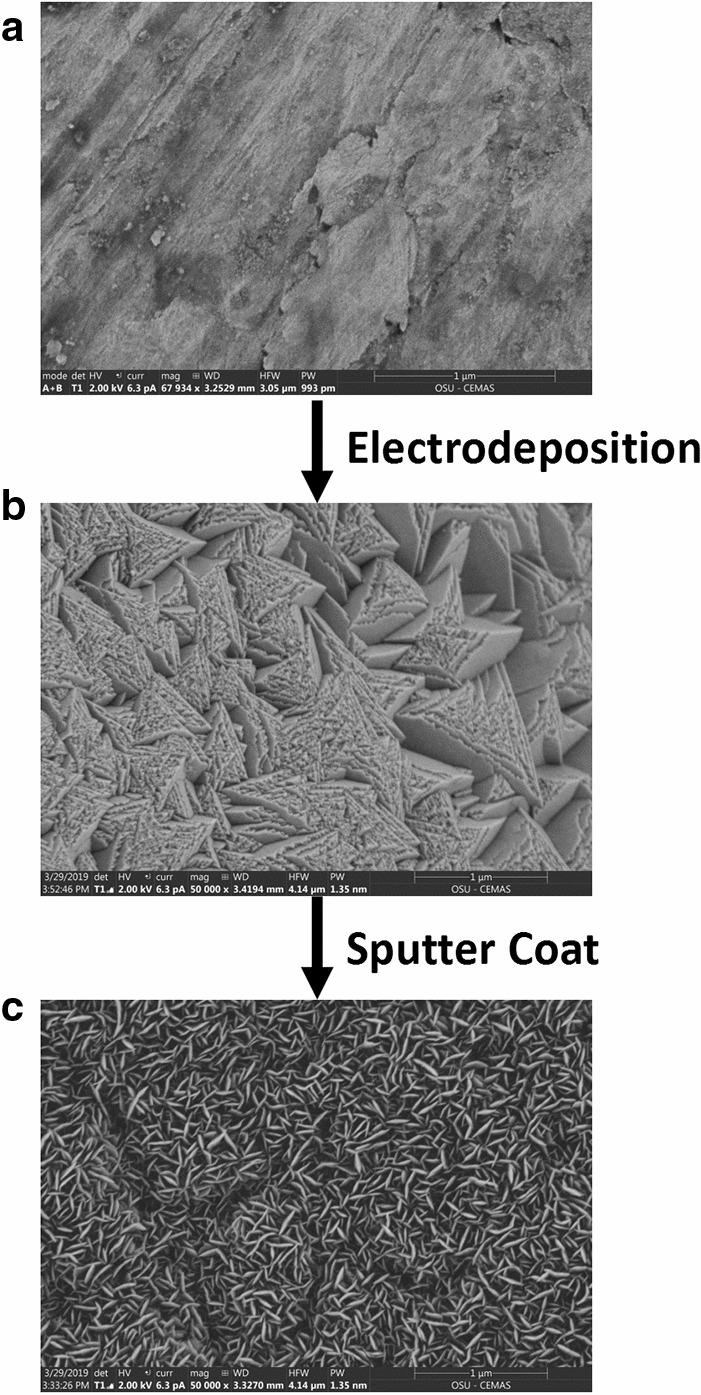
SEM of the cathode surface. (**a**) Bare copper mesh substrate, (**b**) electrodeposited copper oxide on copper mesh, and (**c**) Ni nanolayer as sputter-deposited on copper oxide-copper substrate. Scale bar = 1 μm.

**Figure 2:**
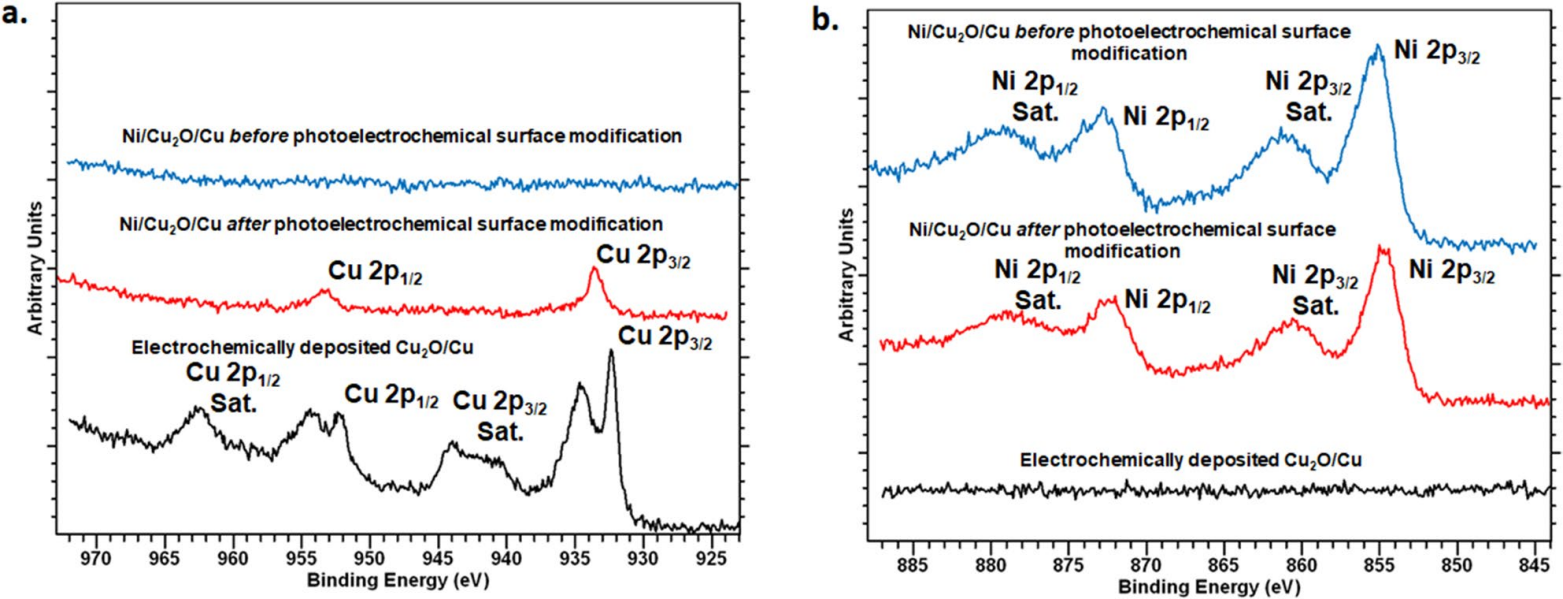
XPS analyses at various stages of the electrode fabrication process. (**a**) XPS spectra of Ni-Cu_2_O-Cu electrode after each step of the fabrication process and after photoelectrochemical surface modification. Main and satellite peaks corresponding to copper and its oxides are annotated. (**b**) XPS spectra of Ni-Cu_2_O-Cu electrode after each step of the fabrication process and after photoelectrochemical surface modification over the range of energies corresponding to nickel; satellite peaks are also marked. The peak between 960 and 965 eV and structure between 940 and 950 eV in the figure on the left is indicative of CuO whereas the other peaks are indicative of Cu_2_O^35^. The split Cu 2p_3/2_ and 2p_1/2_ peaks in (**a**) indicate the presence of a CuO/Cu_2_O mixed phase. Note that after photoelectrochemical surface modification, only the peaks of Cu (0) are evident (middle curve in red in panel (**a**)) and the peaks corresponding to both CuO and Cu_2_O are absent.

Figure 1 shows scanning electron microscope (SEM) images of the changing surface morphology with each successive step of the electrode fabrication process. Cross-section SEM (Supplementary Fig. S3) and X-ray Energy Dispersive Spectroscopic (EDS) analysis (see Supplementary Figs. S4-S5), along with repetition of identical process steps on a copper foil (see Supplementary Figs. S6-S7), indicate that the electrodeposited copper oxide layer is about 4.26-μm thick and the sputtered Ni nanolayer is less than ~ 50 nm. EDS analyses of the surface at various stages of the fabrication process indicate the presence of Cu and O after electrodeposition, with Ni peaks and strong Ni/Cu ratios appearing after sputtering (Supplementary Fig. S5). Cross-sectional EDS maps of the surface suggest complete coverage of the surface with Ni (Supplementary Fig. S4). Despite the visible reddish appearance of the electrode, the surface morphology of the nanostructured heterolayer electrode (Fig. 1c) resembles that of CuO prepared by chemical bath deposition (compare with figs. 6e and 7a–c in ref.^28^). Therefore, it is likely that our electrode comprises both Cu_2_O and CuO but that the exposed surface is primarily CuO. X-ray Photoelectron Spectroscopy (XPS) spectra (Fig. 2 and Supplementary Fig. S8) clearly show the presence of both CuO and Cu (and perhaps Cu_2_O as well) on the surface after electrodeposition and prior to sputtering (bottom curves in Fig. 2)^28,34,35^. These peaks are absent after sputter coating with Ni (top curve in Fig. 2a) and only the 2p_1/2_ and 2p_3/2_ peaks of Ni are evident^34^ (top curve in Fig. 2b). Note that the morphology resembling CuO appears *after* sputtering of the Ni nanolayer (see Fig. 1c versus Fig. 1b), suggesting that what might have been mostly electrodeposited Cu_2_O is converted to CuO after the sputtering step.

### Photoelectrochemical surface modification of the cathode depletes oxygen in the surface layers

**Figure 3:**
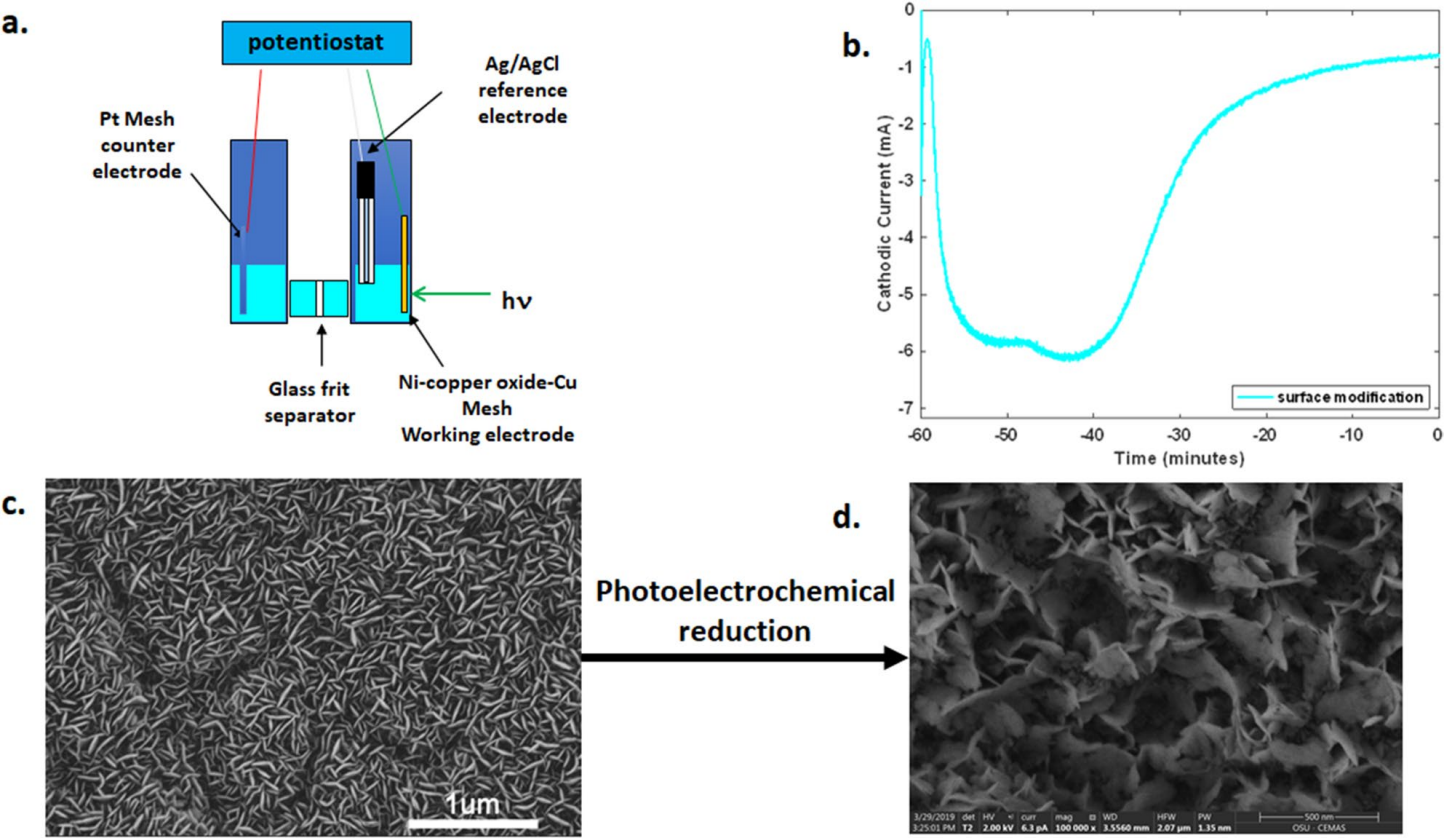
Photoelectrochemical modification of the Ni-copper oxide-Cu surface. (**a**) Schematic of custom quartz H-cell with glass frit separator used for regeneration of NADPH from NADP^+^, (**b**) Cathodic current versus time in the presence of 532 nm laser irradiation in a 0.5 M sodium phosphate (pH 8) with the electrode at − 0.75 V with respect to a Ag/AgCl (3 M NaCl) reference electrode during the earlier portion of the photoelectrochemical surface modification process, (**c**) SEM of Ni-copper oxide-Cu surface before photoelectrochemical surface modification (same as Fig. 1c), (**d**) SEM of Ni/copper-oxide/Cu surface after photoelectrochemical surface modification. Scale bar = 1 μm.

In the final step of electrode fabrication, the Ni-Cu_2_O-Cu electrode [immersed in sodium phosphate (0.5 M, pH 8)] was exposed to 10 mW, 532 nm unfocused laser radiation. This process was conducted in a customized quartz H-cell with a glass-frit separator to minimize light attenuation through the walls of the cell (Fig. 3a). A planar, geometric surface area of ~ 1 cm^2^ (on the side where Ni was sputtered for 96 h) was illuminated for all cathodes. Potentiostatic electrolysis was performed in conjunction with laser illumination in the same apparatus (Gamry Interface 1000, Fig. 3a) with an Ag/AgCl (3 M NaCl) reference electrode and Pt mesh (Alfa Aesar 10283) counter electrode. Figure 3b shows the variation of the cathodic current versus time during the earlier stage of photoelectrochemical reduction. After an hour, surface modification via photoelectrochemical reduction is complete (Supplementary Fig. S9). The accompanying changes in morphology before and after photoelectrochemical surface modification are shown in the SEM images in Fig. 3c,d.

The features characteristic of CuO (Figs. 1c and 3c) have been previously reported in the chemical bath deposition process of ref.^28^. These features change after surface modification of the electrode and are significantly different from their as-deposited form (Fig. 3d versus Figs. 1b and 3c). EDS analysis reveals the presence of elemental O, Ni, and Cu before and after photoelectrochemical surface modification, but the relative amounts of these elements change with O being less prominent compared to Ni and Cu in the surface layers (Supplementary Fig. S10). XPS spectra also reveal that upon photoelectrochemical surface modification, the Ni peaks 2p_1/2_ and 2p_3/2_ peaks still appear prominently (middle curve in Fig. 2b) but only the main 2p_1/2_ and 2p_3/2_ peaks of Cu (middle curve in Fig. 2a) remain, indicating the likely conversion of CuO and Cu_2_O to Cu on the electrode surface^34,35^. Thus, after photoelectrochemical surface modification, the Ni–Cu_2_O–Cu mesh electrode is devoid of the oxide, likely leaving a Ni–Cu surface.

**Figure 4:**
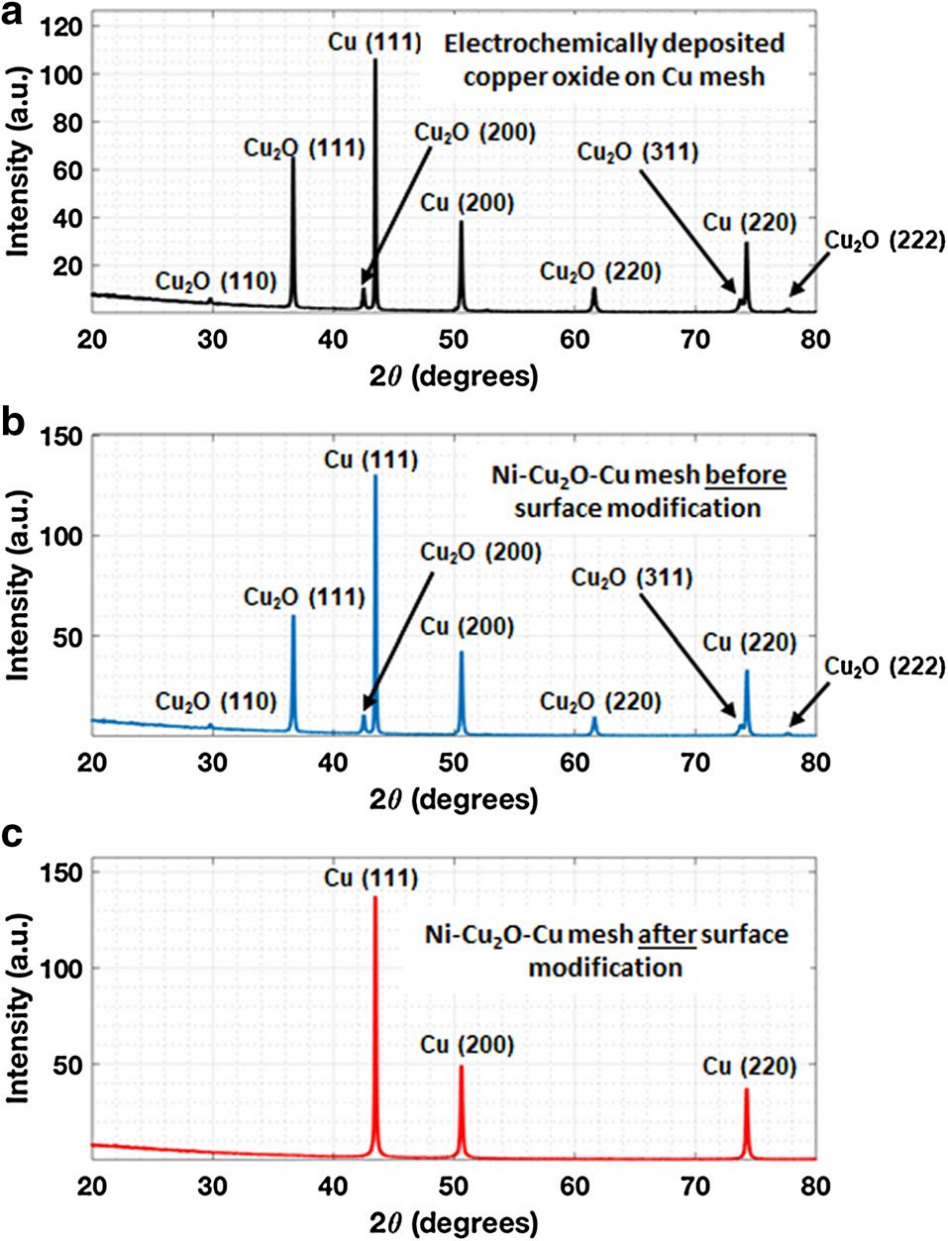
X-ray diffraction (XRD) spectra of electrode surface at various stages of the fabrication process. **(a)** XRD spectra of electrochemically deposited copper oxide on the Cu mesh. **(b)** XRD spectra of the nanostructured heterolayer electrode before photoelectrochemical surface modification. **(c)** XRD spectra of the nanostructured heterolayer cathode after photoelectrochemical surface modification. Note the disappearance of the oxide peaks, consistent with EDS and XPS results. The (111), (200), and (220) peaks of Cu at 2θ = 43.44°, 2θ  = 50.5°, and 2θ = 74.2°, respectively, are evident in all three spectra^36,42–44^.

A cross section of the surface-modified electrode was prepared by focused ion beam (FIB) milling and imaged using the SEM. EDS analysis of the surface layers (Supplementary Figs. S11–S14) reveals the absence of elemental oxygen, supporting the idea that what remains of the surface after photoelectrochemical surface modification is Cu with a Ni nanolayer (< 50 nm thickness). Figure 4 shows X-ray diffraction (XRD) spectra prior to sputtering Ni, after coating with Ni and before photoelectrochemical surface modification, and after photoelectrochemical surface modification. These results confirm photoelectrochemical reduction of the copper oxide to copper since only the peaks corresponding to metallic copper remain in the near-surface layers and those corresponding to the oxide(s) of copper disappear^36^.

### The cofactor NADPH is directly electrochemically regenerated from NADP^+^ at low overpotential

**Figure 5:**
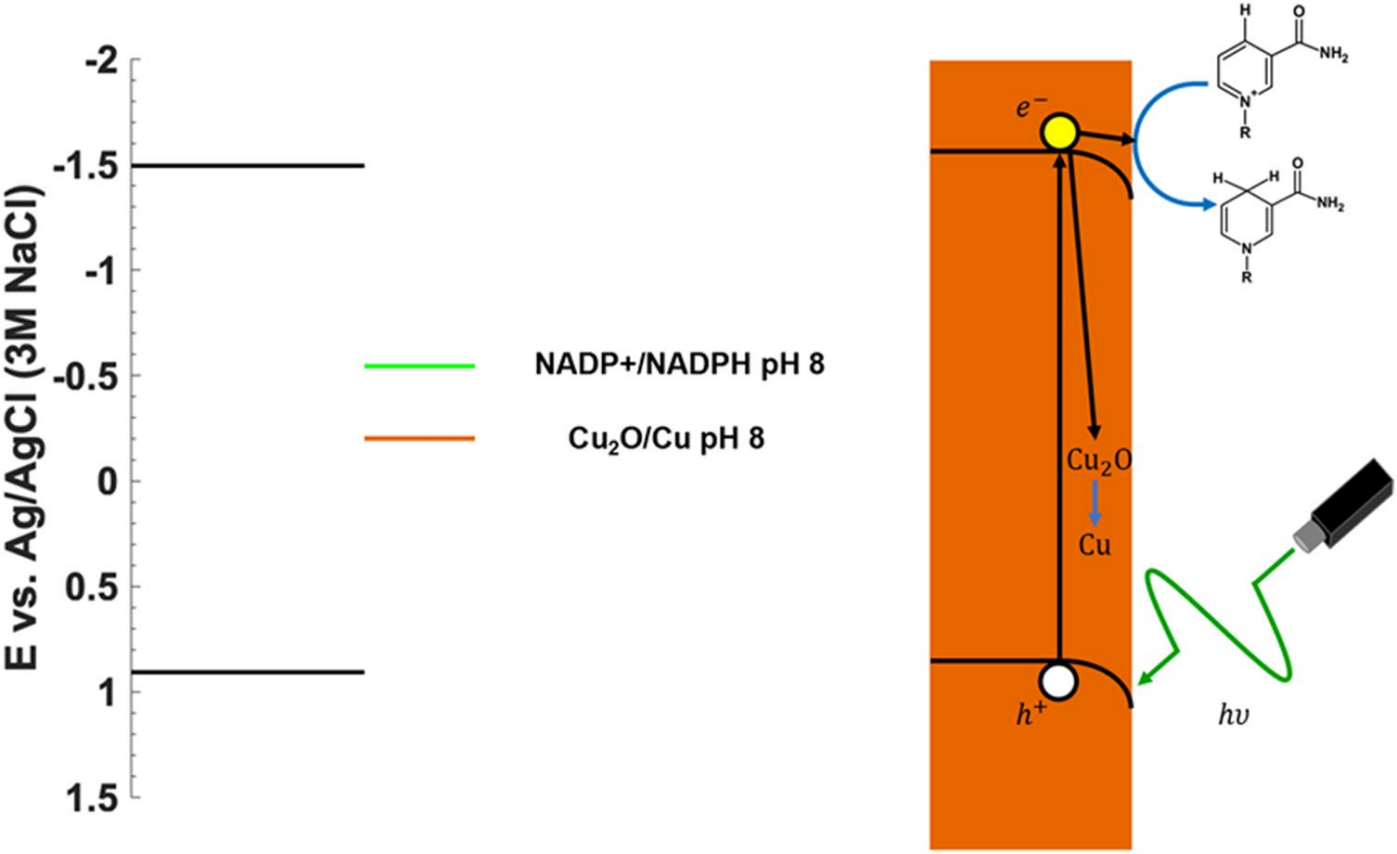
Band energy diagram. Schematic indicating redox potentials for NADP^+^ and Cu_2_O reduction at pH = 8 relative to Ag/AgCl (3 M NaCl). The nickel layer, necessary for providing adsorbed hydrogen, is not shown.

Figure 5 shows a schematic of the electrochemical process and corresponding band-energy diagram for NADPH regeneration used here. The redox potentials for NADP^+^/NADPH and Cu_2_O/Cu at pH 8 are shown (Fig. 5; Supplementary Eq. S2). When photons of sufficient energy are absorbed by copper oxide electrons that are promoted to the conduction band. When these electrons do not recombine with holes, they are available for electrochemical reactions subject to an electric field in the depletion layer. Since the redox potential for Cu_2_O/Cu is more positive than for NADP^+^/NADPH, the photoelectrochemical reactions occurring during electrode surface modification likely lead to formation of Cu from copper oxide, and thus generate a Ni–Cu surface from the original Ni–Cu_2_O–Cu surface layer. We show here that photoelectrochemical processing may be used as surface modification either prior to or in tandem with cofactor regeneration.

Bulk electrolyses of 1.5 mM NADP^+^ solutions (Sigma 10128031001) were performed in the same apparatus used for photoelectrochemical surface modification of the cathode and with the same buffer (Fig. 3a). All experiments were conducted by applying a fixed electrode potential of − 0.75 V (with respect to Ag/AgCl (3 M NaCl)) to the nanostructured heterolayer cathode. The *LbADH* assay was then used to determine the purity and activity of the reaction products (see “Methods”). The enzyme *Lb*ADH catalyzes the NADPH-mediated reduction of butyraldehyde to butanol. The enzyme *Lb*ADH will only accept the active isomer 1,4-NADPH as a cofactor along with the aldehyde substrate (butyraldehyde). This assay is selective, and the conversion of butyraldehyde to butanol will not proceed with either inactive isomers such as 1,6-NADPH or the (NADP)_2_ dimer. For determination of utility of the product, 350-μL aliquots were withdrawn from the cathode side of the chamber (Fig. 3a) where the cofactor is regenerated. The characteristic absorbance of NADPH at 340 nm was monitored (see sample absorption spectrum in Supplementary Fig. S15) to determine the presence of any NADPH derivatives that are produced during the reaction. Upon initiating the aldehyde reduction, any observed decrease in absorbance at 340 nm is solely due to the oxidation of 1,4-NADPH because of the assay’s selectivity; any residual absorbance at 340 nm after termination of the reaction is attributable to enzymatically inactive products [e.g. 1,6-NADPH, (NADP)_2_].

**Figure 6:**
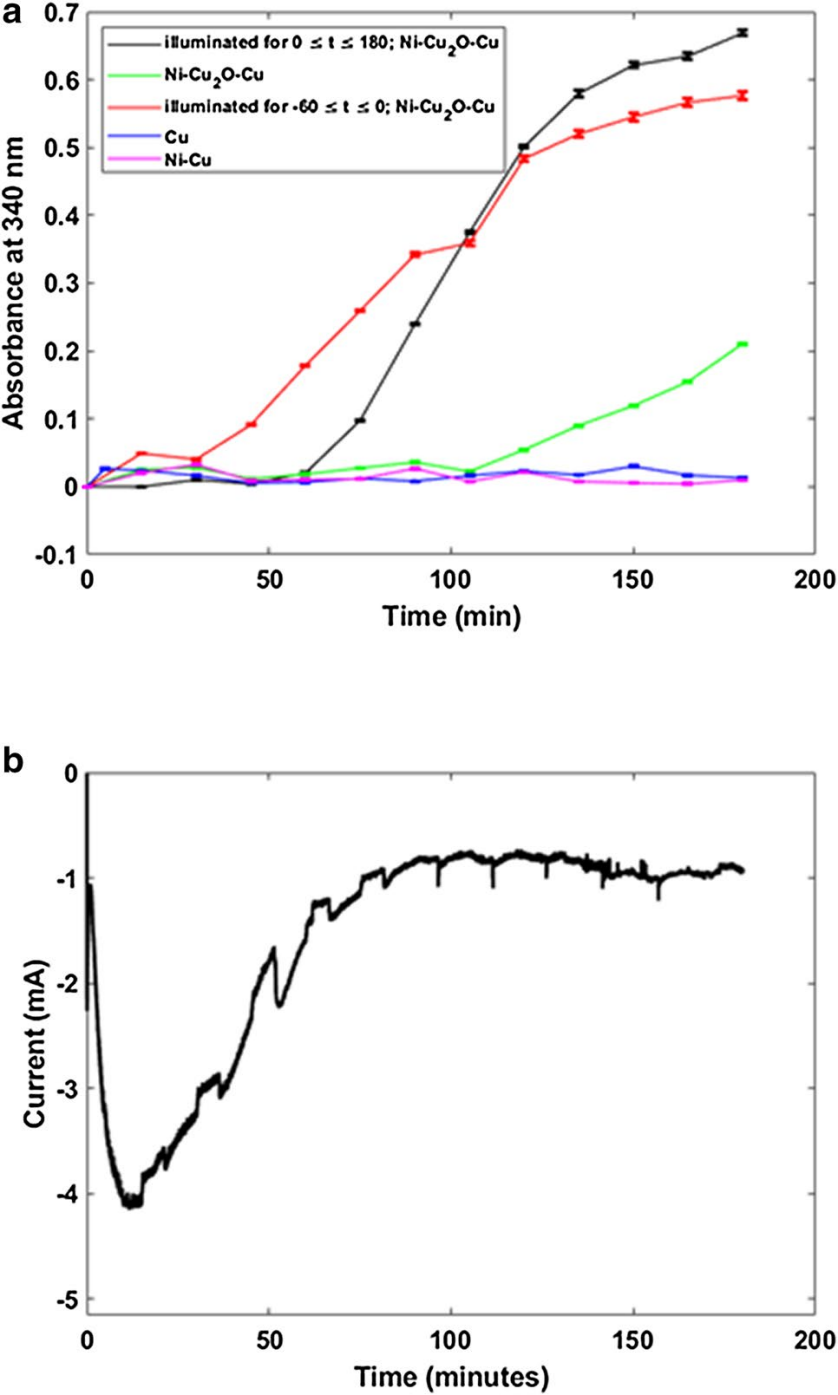
Cofactor regeneration with different cathode materials and conditions of illumination. (**a**) Absorption at 340 nm for NADPH (product) versus time for electrochemical reduction in the presence of illumination for the entire duration of the experiment with the nanostructured heterolayer electrode (black curve); illumination of the nanostructured heterolayer cathode first for 60 min, followed by electrochemical reduction (red); electrochemical reduction with a non-modified nanostructured heterolayer electrode in the absence of illumination (green); electrochemical reduction with the Cu mesh alone and in the absence of illumination (dark blue); and electrochemical reduction with Ni sputtered on the Cu mesh alone and in the absence of illumination (magenta). The time point t = 0 represents the instant when electrochemical reduction is initiated. Error bars represent the standard deviation of 30 sequential absorbance measurements on the same aliquot. (**b**) Current trace versus time for the nanostructured heterolayer (as fabricated and not surface modified) electrode in the presence of both 532 nm (10 mW) laser illumination and electrochemical reduction.

Figure 6a shows a time-course study of the absorbance of NADPH at 340 nm for various cathodic materials that were subject to illumination and not, where t = 0 indicates initiation of electrochemical reduction of NADPH. All reactions were initiated with 1.5 mM NADP^+^. Cofactor regeneration only occurs for the oxide-derived cathodes as no regeneration is observed for either pure Cu cathodes or electrodes with Ni sputtered directly on a Cu substrate. Illumination with a low-power (10 mW), 532-nm unfocused laser irradiation accelerates the initiation of cofactor regeneration compared to electrochemical regeneration alone. Based on the time-course results (Fig. 6a, it is evident that laser irradiation *only* modifies the electrode surface to enable subsequent electrochemical cofactor regeneration (black, red, and green curves in Fig. 6a; absence of NADPH indicated by lack of absorption at 340 nm). Moreover, in the absence of photoelectrochemical surface modification and any illumination, electrochemistry alone supports NADPH regeneration, albeit at a much slower rate. From our time-courses for cofactor regeneration by electrochemistry alone (green curve in Fig. 6a), we are unable to determine whether the level of NADPH regeneration is comparable to the more rapid photoelectrochemical regeneration (black and red curves in Fig. 6a).

Figure 6b shows the variation of current (cathode at − 0.75 V with respect to Ag/AgCl reference) for the case where a Ni–Cu_2_O–Cu mesh electrode that has not previously undergone photoelectrochemical surface modification, is used to photoelectrochemically regenerate NADPH *in the presence* of the same 532 nm laser irradiation used for surface modification of other Ni-Cu_2_O-Cu cathodes (Fig. 6b). The similarity between Figs. 6b and 3b indicates that the effect of laser irradiation is exclusively to modify the surface photoelectrochemically as no cofactor regeneration is observed during the first ~ 60 min in any of the experiments involving photoelectrochemical conversion (Fig. 6a). In contrast, electrochemical regeneration of NADPH with cathodes that have previously undergone photoelectrochemical surface modification (red curve in Fig. 6a) is just as effective with respect to the amount regenerated compared to those experiments where laser irradiation is simultaneous with electrochemical regeneration. The notable difference is the onset time for the NADPH product.

### The regenerated cofactor NADPH does not contain the inactive dimer

We investigated the purity of the product of electrochemical regeneration (NADPH) using the *Lb*ADH enzyme (Methods)^31^. When this enzyme was added to commercially obtained NADPH (60 μM) in the presence of butyraldehyde, we observed near-complete conversion to NADP^+^ (Q > 99%, Supplementary Eqs. (S3–S5), Supplementary Fig. S16). Direct electrochemical regeneration using the photoelectrochemically surface-modified cathodes, however, showed that only 66% could be converted to NADP^+^. This result suggests that direct cofactor regeneration using the nanostructured heterolayer Ni–Cu_2_O–Cu mesh cathodes result in the formation of some inactive NADPH or products. Since it is well established that electrochemical regeneration of NADH from NAD^+^ results in unwanted products such as the inactive dimer^1, 7,10,13,37,38^, we sought to investigate this possibility.

**Figure 7:**
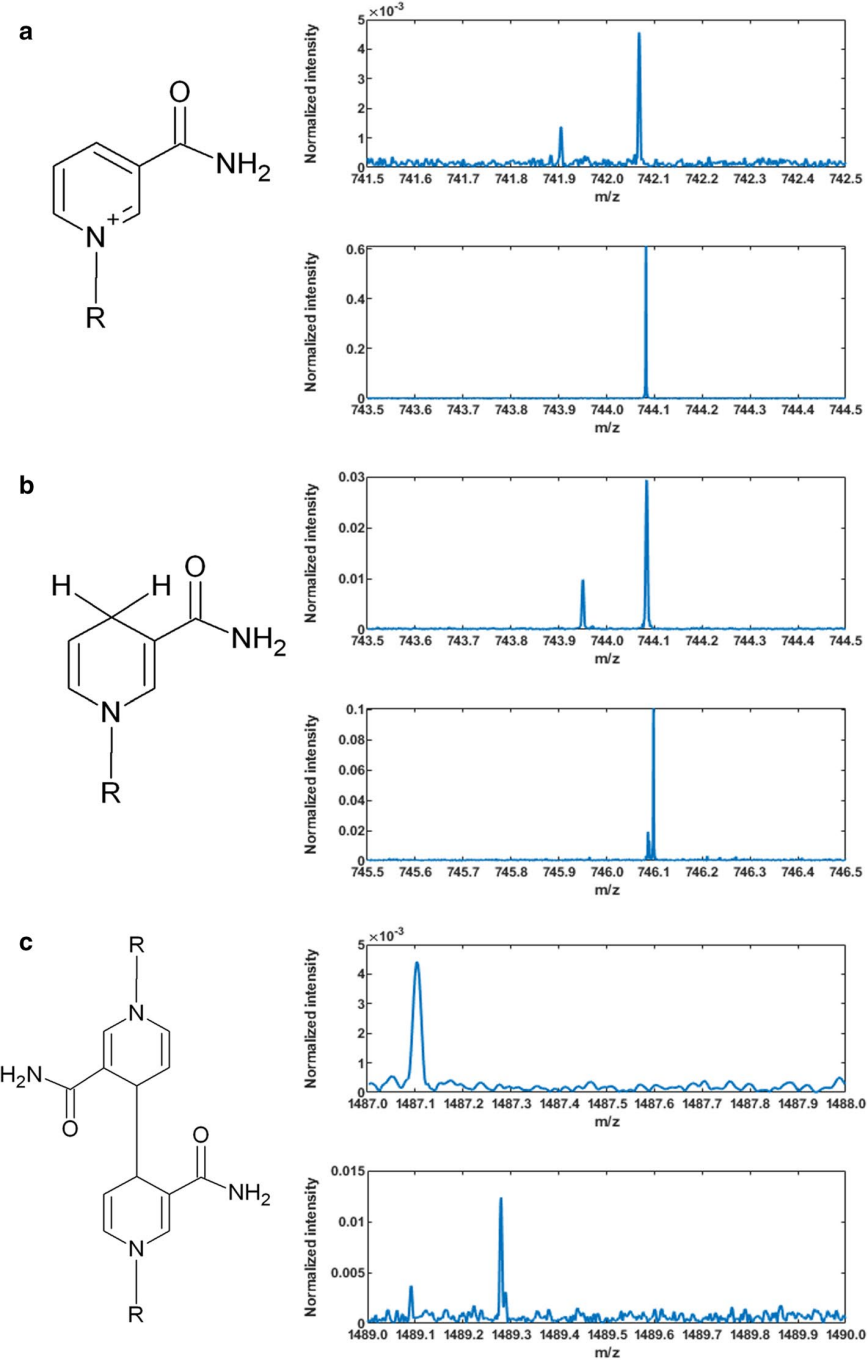
High-resolution FT-ICR MALDI spectra of products of NADPH electrochemically regenerated with photoelectrochemically surface modified Ni–Cu_2_O–Cu cathode. Mass-to-charge (m/z) ratios corresponding to NADP^+^ **(a)**, NADPH **(b)**, and (NADP)_2_ dimer **(c)** in the product. For each molecule, the top plot represents spectra obtained in negative-ion mode and the bottom plot represents spectra obtained in positive-ion mode. Presence of singly ionized NADP^+^ and NADPH is indicated in both positive- and negative-ion modes. No strong peaks were observed for singly ionized dimer in either positive- or negative-ion mode.

Fourier-transform ion cyclotron resonance mass spectrometry (Bruker 15 T FT-ICR MS) with matrix-assisted desorption/ionization (MALDI) in both negative- and positive-ion modes was used to determine the composition of the products of cofactor regeneration. We used α-cyano-4-hydroxy-cinnamic acid as the matrix. This instrument is capable of ultrahigh resolution (> 10^6^) and mass accuracy (< 1 ppm). Figure 7 shows FT-ICR MS spectra in both negative-ion mode (top figures in Fig. 7a–c) and positive-ion mode (bottom figures in Fig. 7a–c). The amplitudes in Fig. 7 are qualitative indicators only since the abundance of a compound is determined by its ionization efficiency. Singly ionized NADP^+^ and NADPH are present in both positive- and negative-ion modes. No strong peaks are observed for the singly-ionized dimer in either positive- or negative-ion mode, indicating that its presence is negligible. The FT-ICR MS data confirm conversion of NADP^+^ to NADPH, consistent with the results of the *Lb*ADH assay. A negligible amount of the dimer, (NADP)_2_, is formed in this direct electrochemical regeneration of the cofactor in stark contrast to previous reports^13,38^. The FT-ICR MS MALDI results also suggest that the remaining one-third of the product that cannot support the redox reaction as determined by the *Lb*ADH assay could be an inactive isomer (Supplementary Fig. S1) that cannot be reliably parsed from the active version by FT-ICR MS MALDI.

The above results highlight the favorable cofactor regeneration outcome obtained with the unexpected morphology and properties of the oxide-derived copper-nickel (odCu-Ni) surface, produced by photoelectrochemical surface modification of the nanostructured heterolayer Ni-Cu_2_O-Cu cathode. We propose a mechanism based on the results presented here:

1 $$\left(odCu-Ni\right)+{H}_{2}O+{e}^{-}\rightleftarrows \left(odCu-Ni\right)+{H}_{ads}+{OH}^{-}$$

2 $$\left(odCu-Ni\right)+{NADP}^{+}\rightleftarrows \left(odCu-Ni\right)+{NADP}_{ads}^{+}$$

3 $$\left(odCu-Ni\right)+{NADP}_{ads}^{+}+{e}^{-}\rightleftarrows \left(odCu-Ni\right)+{NADP}_{ads}^{*}$$

4 $$\left(odCu-Ni\right)+{NADP}_{ads}^{*}+{H}_{ads}\rightleftarrows \left(odCu-Ni\right)+{NADPH}_{ads}$$

5 $$\left(odCu-Ni\right)+{NADPH}_{ads}\rightleftarrows \left(odCu-Ni\right)+NADPH$$

where the subscript *ads* denotes a species adsorbed on the odCu-Ni surface, and the superscript * denotes an excited state. The first step (Eq. 1) is simply the commonly observed Volmer-type mechanism for adsorption of H on Ni^33^. The steps indicated in Eqs. 2–4 represent hydrogenation of the adsorbed NADP^+^ with an adsorbed H atom before its radicalization by an electron provided by the electrode. The resulting NADPH is then desorbed back into the electrolyte (Eq. 5). Alternatively, Eqs. 3–4 may be replaced by:

6 $$\left(odCu-Ni\right)+{NADP}_{ads}^{+}+{H}_{ads}\rightleftarrows \left(odCu-Ni\right)+{NADPH}_{ads}^{*+}$$

7 $$\left(odCu-Ni\right)+{NADPH}_{ads}^{*+}+{e}^{-}\rightleftarrows \left(dCu-Ni\right)+{NADPH}_{ads}$$

In this instance, NADPH subsequently desorbs according to Eq. 5. These reaction pathways could all equally lead to formation of the 1,6-NADPH inactive isomer. The inactive dimer was not detected in the FT-ICR MS MALDI spectra. These two results together indicate that the inactive product is likely the isomer which could form adjacent to the cathode sheath where higher electric fields are expected.

## Discussion

We have developed a nanostructured Ni–Cu_2_O–Cu heterolayer material and demonstrated photoelectrochemical regeneration of NADPH. Sputtering of nickel on a copper oxide electrode produced an unexpectedly desirable surface morphology leading to high product selectivity. The demonstrated properties of this cathode for direct electrochemical regeneration of NADPH are at the lowest reported overpotential (− 0.75 V versus Ag/AgCl (3 M NaCl) reference). Material characterization by SEM, EDS, XPS, and XRD all confirm that sputtering of Ni on the heterolayer Cu_2_O–Cu mesh cathode leads additionally to formation of CuO in the surface nanolayers. Furthermore, after photoelectrochemical surface modification, oxygen is depleted from the surface layers of the electrode to generate a Ni–Cu nanolayer. The NADPH product electrochemically regenerated from NADP^+^ using this nanostructured heterolayer cathode is found to be free of any dimers. This finding contrasts with previous studies that employed more expensive electrode materials^13,15,38,39^ or even a Cu mesh electrode with the same sputtered Ni overcoat (this work).

The concomitant ability of the cathode to adsorb a hydrogen and donate an electron to NADP^+^ appears to greatly diminish the propensity of the radicalized NADP^+^ to form an inactive (NADP)_2_ dimer. Despite the unexpected payoffs from this electrode material, our *Lb*ADH enzyme assay revealed the presence of another inactive product, possibly an isomer such as 1,6-NADPH that may be generated in the electrode-adjacent layers in the electrochemical H-cell. Previous work^13^, as with our study, utilized static (non-flowing) media, which could account for the accumulation of inactive products. In practice, production of the unwanted inactive isomer could be suppressed by using a flow cell rather than static or batch processes. Recent work on electrochemical regeneration of the non-phosphorylated cofactor NADH, using multiwalled carbon nanotubes grown on a stainless steel mesh and decorated with nickel nanoparticles, showed a recovery of 98% but at elevated cathode potentials of − 1.168 V (vs. Ag/AgCl [3 M NaCl])^39^. An optimum potential higher than − 0.75 V, which we used in this work with our nanostructured heterolayer Ni-Cu_2_O-Cu electrode, may produce higher yields of active NADPH may therefore exist, and should be explored further.

Beyond significantly advancing cofactor regeneration, the electrode material and associated photoelectrochemical surface modification process reported here could advance processes for water-splitting for hydrogen generation, artificial photosynthesis^29^, and synthesis of biofuels such as butanol via fermentation^12,40,41^.

## Methods

### Spectrophotometric measurements

All spectrophotometric measurements were carried out with a Thermo Scientific Evolution 300 UV–Vis spectrophotometer using the VisionPro software. All measurements were conducted in fixed wavelength mode with an integration time of 3 s and a wavelength bandwidth of 1 nm. Samples were pipetted into 10 mm pathlength micro-quartz cuvettes (Fisher) and mixed by inversion prior to measurement. Errors were determined from the standard deviation of 30 sequential absorbance measurements on the same aliquot, for each time point.

### *Lb*ADH assay to determine the turnover number

The *Lb*ADH activity assay was performed as described elsewhere^31^, albeit with some modifications. All assays were carried out at 37 °C in a 30-μl reaction volume. Typically, the reaction mix contained 5 mM butyraldehyde, 0.25 mM NADPH in 50 mM potassium phosphate (pH 8). The reaction was initiated by the addition of 1.5 μl of 0.29 μM recombinant ADH to a 28.5-μl reaction mix. From the 30-μl reaction that was assembled, 28-μl was immediately transferred to a 384-well microplate and the absorbance at 340 nm was monitored real-time using a SpectraMax M5 (Molecular Devices) Microplate Reader (integration time of 1000 ms; settle time of 300 ms). This continuous spectrophotometric readout enabled calculation of initial velocities. Linear regression analysis of NADPH generated as a function of time was used to calculate the initial velocity (0.97 ≤ r^2^ ≤ 0.99). For each assay, a control assay was performed that included all the assay components except the ADH. Using the initial velocities determined under saturating butyraldehyde (5 mM), a k_cat_ of 214 ± 5 min^−1^ was determined from three assay replicates. This turnover number is consistent with the value reported earlier^31^.

### *Lb*ADH assay for detecting electrochemically regenerated NADPH

The *Lb*ADH enzyme assay for determination of NADPH selectivity in electrochemical regeneration was performed by first collecting a t_0_ aliquot of NADP^+^ solution (1.5 mM, 325 μL) prior to initiating the time-course. This sample was then mixed with 13 μL 0.25 M butyraldehyde and diluted with potassium phosphate buffer solution (pH 8) to yield a final concentration of 9.3 mM butyraldehyde in a total volume of 350 μL. This butyraldehyde concentration was chosen to ensure adequate amount of substrate for complete turnover of all regenerated 1,4-NADPH. The A_340_ measurement of this sample served as a reference for baseline subtraction (A_ref_). Three-hundred and twenty-five μL of regenerated cofactor was then collected and mixed with 13 μL 0.25 M butyraldehyde substrate and its initial absorbance at 340 nm (A_0_) was recorded. Twelve μL 58 μM *Lb*ADH was then added to the regenerated cofactor/butyraldehyde mixture to yield a final concentration of ~ 2 μM ADH, and its absorbance at 340 nm was monitored until a steady state was reached (A_f_). The selectivity, Q, of the sample was calculated according to Eq. 1, where the constant, $\alpha$, represents the factor by which the regenerated cofactor/butyraldehyde mixture is diluted upon addition of *Lb*ADH:

8 $$Q=1-\frac{{(A}_{f}-{A}_{ref})}{\alpha ({A}_{0}-{A}_{ref})}$$

Inclusion of the constant in this equation accounts for any decrease in absorbance upon addition of *Lb*ADH solely by virtue of dilution. To ensure applicability of Beer’s law, only regenerated cofactor samples with an initial absorbance less than 0.25 (~ 70 μM NADPH) were considered (Supplementary Fig. S17). Below this threshold, the absorbance-concentration behavior of 1,4-NADPH in sodium phosphate buffer (pH 8) was observed to be approximately linear.

## Supplementary Information

Supplementary Information
