# Supplementary material for: Copper oxide-based cathode for direct NADPH regeneration
Source: Sci Rep. 2021 Jan 8;11:180. doi: 10.1038/s41598-020-79761-6 (PMC7794519; doi:10.1038/s41598-020-79761-6)
Supplement: Supplementary file 1 — Supplementary Information [file 41598_2020_79761_MOESM1_ESM.pdf]

Supplementary Information

Copper Oxide Based Cathode for Direct NADPH Regeneration

J. Kadowaki, T. H. Jones, A. Sengupta, V. Gopalan, and V. V. Subramaniam

Contents:

Supplementary Methods
Supplementary Figures S1-S17

Calculation of redox potentials

The electrodeposition reaction used for depositing copper oxide on the copper mesh is given by:

$$2CuL_2 + 2OH^- + 2e^- \rightarrow Cu_2O + H_2O + 4L^- \quad \text{(S1)}$$

where L represents the lactate anion. All redox potentials at pH 8 are computed via the Nernst equation:

$$E = E^0 + \frac{2.303RT}{nF} \log_{10} \frac{\prod_{ox} c_{ox}^{\gamma_{ox}}}{\prod_{red} c_{red}^{\gamma_{red}}} \quad \text{(S2)}$$

In equation S2, E^0 is the standard reduction potential of the redox couple, R is the universal gas constant, T is the temperature, n is the number of electrons involved in the half-reaction, F is Faraday's constant, c_{ox} and c_{red} are the concentrations of oxidized and reduced species, respectively, and γ_{ox} and γ_{red} are the stoichiometric coefficients of oxidized and reduced species, respectively. All non-pH determining species, e.g. Cu_2O or NADP+, are considered to be at unit concentrations. The standard reduction potential of the Cu_2O/Cu couple was taken to be -0.360 V (Standard Hydrogen Electrode) and the formal potential (pH 7) of the NADP+/NADPH couple was taken to be -0.320 V (Standard Hydrogen Electrode).

Calculation of selectivity of product, Q

The selectivity, Q, of cofactor regeneration products given by Eq. 1 may be re-written as follows.

$$Q = \frac{\alpha\left(A_0 - A_{ref}\right) - \left(A_f - A_{ref}\right)}{\alpha\left(A_0 - A_{ref}\right)} \quad \text{(S3)}$$

The numerator in Eq. S3 is the decrease in absorbance following the butyraldehyde reduction reaction with *lb*ADH, which is the absorbance due to 1,4-NADPH only. The denominator represents the absorbance of all cofactor regeneration products with non-negligible absorbance at 340 nm excitation, i.e. 1,4-NADPH, $(NADP)_2$ and isomers such as 1,6-NADPH. Invoking the Beer-Lambert Law and denoting the extinction coefficient as ε and concentrations as $[\cdot]$, Eq. S3 takes the following form.

$$Q = \frac{\varepsilon_{1,4-NADPH} l[1,4 - NADPH]}{\varepsilon_{1,6-NADPH} l[1,6 - NADPH] + \varepsilon_{(NADP)_2} l[NADP_2] + \varepsilon_{1,4-NADPH} l[1,4 - NADPH]} \quad \text{(S4)}$$

Finally, if the extinction coefficients are taken to be approximately equal, the selectivity is the ratio of the concentration of enzymatically active 1,4-NADPH to the sum total of the concentrations of cofactor regeneration products.

$$Q \cong \frac{[1,4 - NADPH]}{[1,6 - NADPH] + [NADP_2] + [1,4 - NADPH]} \quad \text{(S5)}$$

Overexpression and purification of *Lb*ADH enzyme

Escherichia coli BL-21 (DE3) cells were transformed with pACYC-*Lb*ADH. A single bacterial colony was used to inoculate 2.5 mL of LB medium supplemented with 35 µg/ml chloramphenicol and grown overnight at 37°C with shaking. This overnight seed culture was used to inoculate 250 mL of fresh LB medium containing the appropriate antibiotics as mentioned above. These cells were grown at 37°C with shaking until the OD_{600} reached 0.6 and were induced with 1 mM IPTG for an additional 3 h. Following IPTG induction, the cells were harvested by centrifugation and the cell pellets were stored at -80°C until further use.

Purification of *Lb*ADH was achieved using immobilized metal-affinity chromatography (IMAC). A 250-mL cell pellet obtained after overexpression, was thawed on ice, re-suspended in 16 mL lysis buffer [95% buffer A, (50 mM Tris-HCl, pH 7.5, 150 mM NaCl, 1 mM DTT); 5% buffer B, (buffer A + 500 mM imidazole)] containing 80 µl bacterial protease arrest (G Biosciences, USA) and sonicated (2s on 5s off, 50% amplitude). After centrifugation of the crude lysate (24,000 g, 30 min, 4°C), the supernatant was applied to 1-mL of 50% slurry of nickel-Sepharose resin (50% slurry) (Nickel Sepharose 6 fast flow, GE Healthcare, Sweden) that had been pre-equilibrated with 5 mL equilibration buffer (95% buffer A + 5% buffer B, without DTT) and mixed gently by nutating at 24°C for 10 min. The resin was allowed to settle down and the supernatant was collected and labeled as the flow-through. The unbound proteins were removed by mixing the resin with 10 ml wash buffer [90% buffer A + 10% buffer B] for 5 min and allowing the resin to settle. The supernatant was collected and labeled as the wash fraction. After washing, the ADH protein was eluted in five successive elution steps each with 0.5 ml elution buffer with increasing imidazole concentration (100 to 500 mM). At each elution step, the resin was mixed with 0.5 mL elution buffer and the clear supernatant after centrifugation was collected separately to serve as the elution fraction. The purity of each fraction was checked by SDS-PAGE analysis and fractions primarily containing ADH were pooled together. The pooled fractions were passed through a SpinX column to remove any adventitiously co-eluted Ni-Sepharose beads and the flow-through was subjected to dialysis for 16 h at 4°C 20 mM Tris-HCl (pH 7.5), 150 mM NaCl, and 1 mM DTT. The concentration of the final protein was determined using its molar extinction coefficient (20,065 $M^{-1}cm^{-1}$) at 280 nm and the final preparation in 10% glycerol was stored at -80°C in small aliquots for subsequent biochemical enzyme assays. From a 250-mL culture, we obtained ~13 mg of recombinant ADH.

SUPPLEMENTARY FIGURES

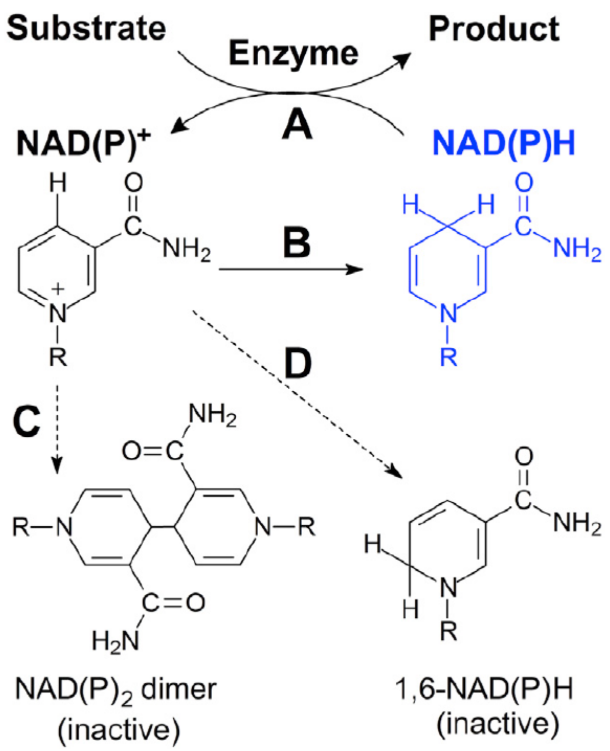

Supplementary Fig. S1: Possible products from regeneration of NADPH. This is Fig. 2 from ref.[10] and is reproduced here with no changes, for ease of reading; **A** depicts a general biocatalyzed reaction showing NADPH being oxidized as a product is formed; **B** shows the desirable pathway for cofactor regeneration; **C** is the pathway for formation of the inactive dimer; **D** is the pathway for forming the inactive isomer.

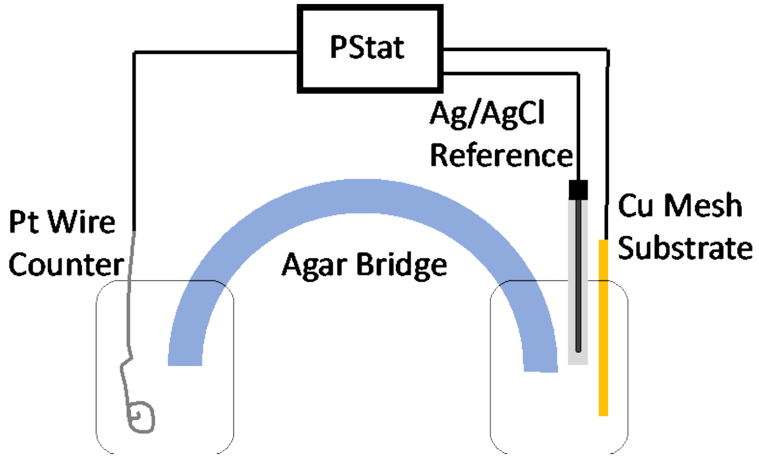

Supplementary Fig. S2: Schematic of two-compartment cell with an agar bridge used to electrodeposit copper oxide on a Cu mesh electrode. Ten-mL beakers were used for both working and counter-electrode compartments. Cupric lactate solution was used in the working electrode compartment for electrodeposition (right) and potassium phosphate buffer (pH 7) was used in the counter-electrode compartment (left).

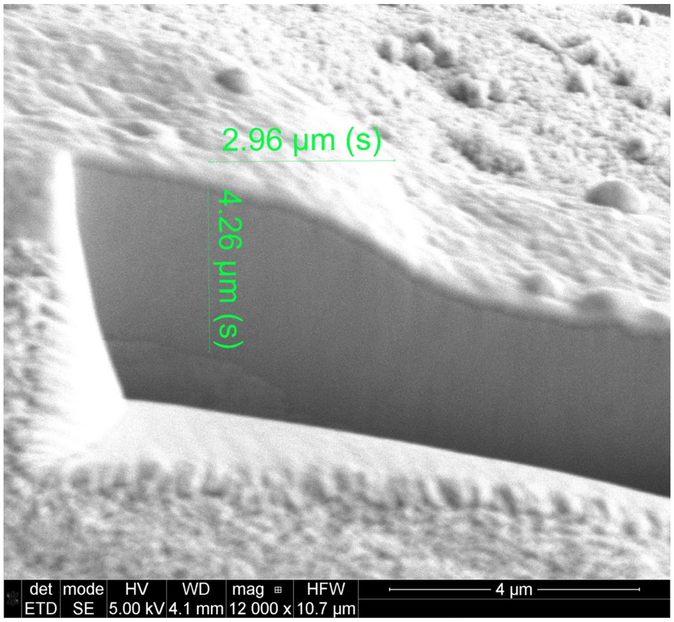

Supplementary Fig. S3: Cross-section FIB-SEM image of Ni-Cu_2O-Cu electrode, after Ni sputter coat and <u>before</u> photoelectrochemical surface modification. The copper oxide layer can be seen to be approximately 4.26 μm thick, with the Ni layer not discernible and likely present under the Pt cap used for SEM imaging. The vertical scale bar is different from the horizontal scale bar because of the tilt of the image. The SEM is capable of accounting for the tilt in measuring distances.

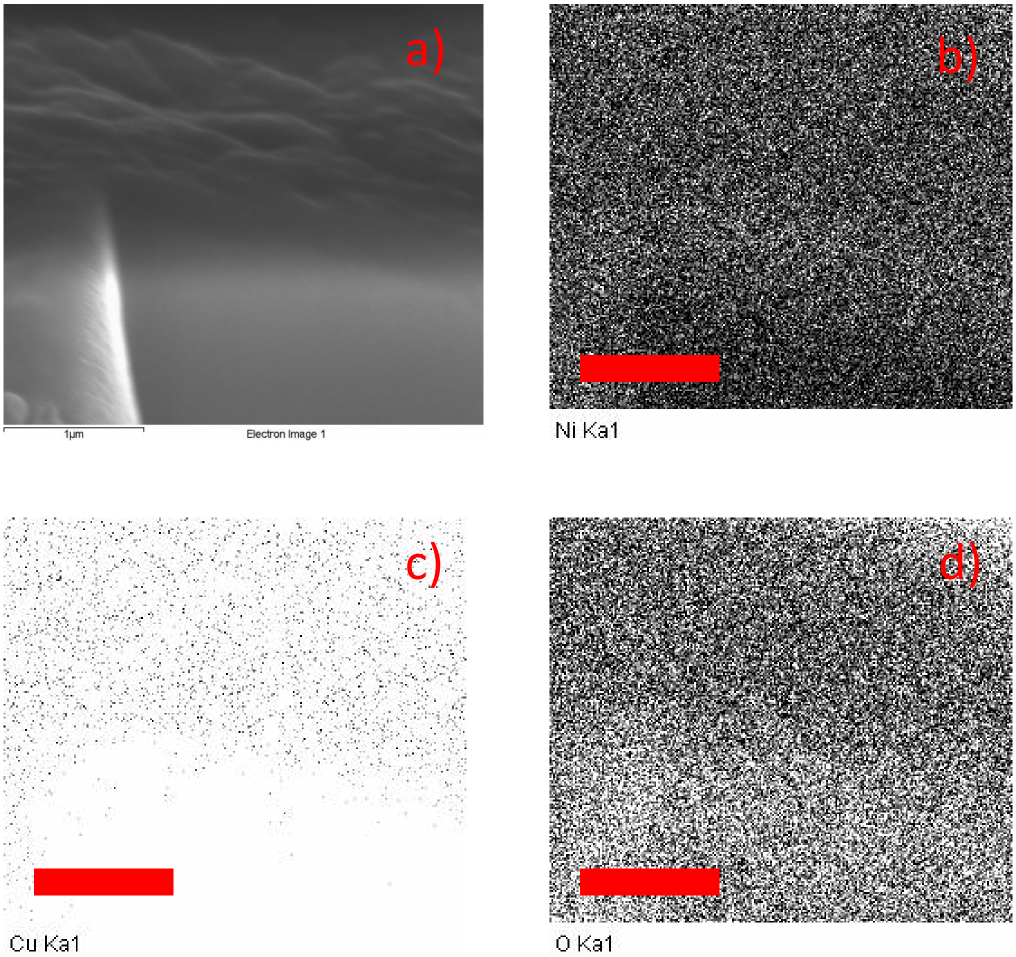

Supplementary Fig. S4: Cross-section EDS maps of elemental Ni, Cu, and O on the Ni/Copper Oxide/Cu electrode <u>before</u> photoelectrochemical surface modification. The sample shown here is the same as that in **Supplementary Fig. 3**. Scale bars are all 1 μm. Pixel intensity is indicative of greater counts for that element (i.e. darker denotes absence while lighter denotes presence).

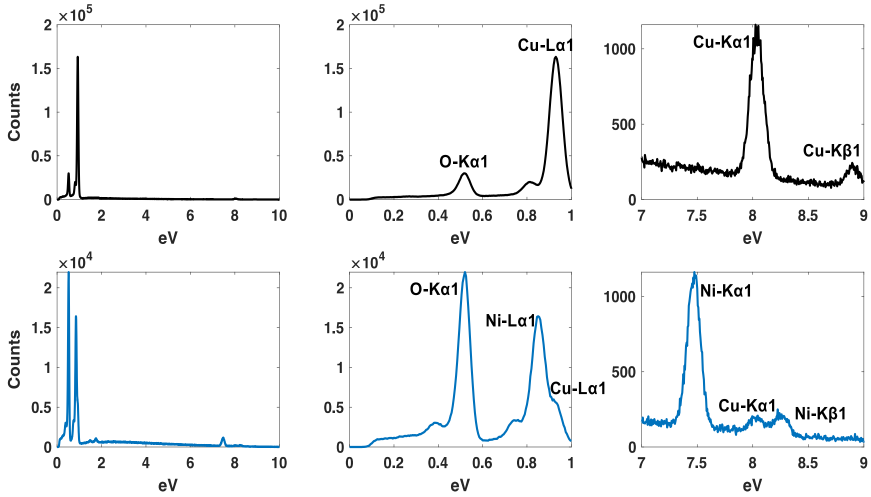

Supplementary Fig. S5: EDS spectra of as-deposited copper oxide (top row) and the Ni-Cu_2O-Cu electrode (bottom row) before photoelectrochemical surface modification.

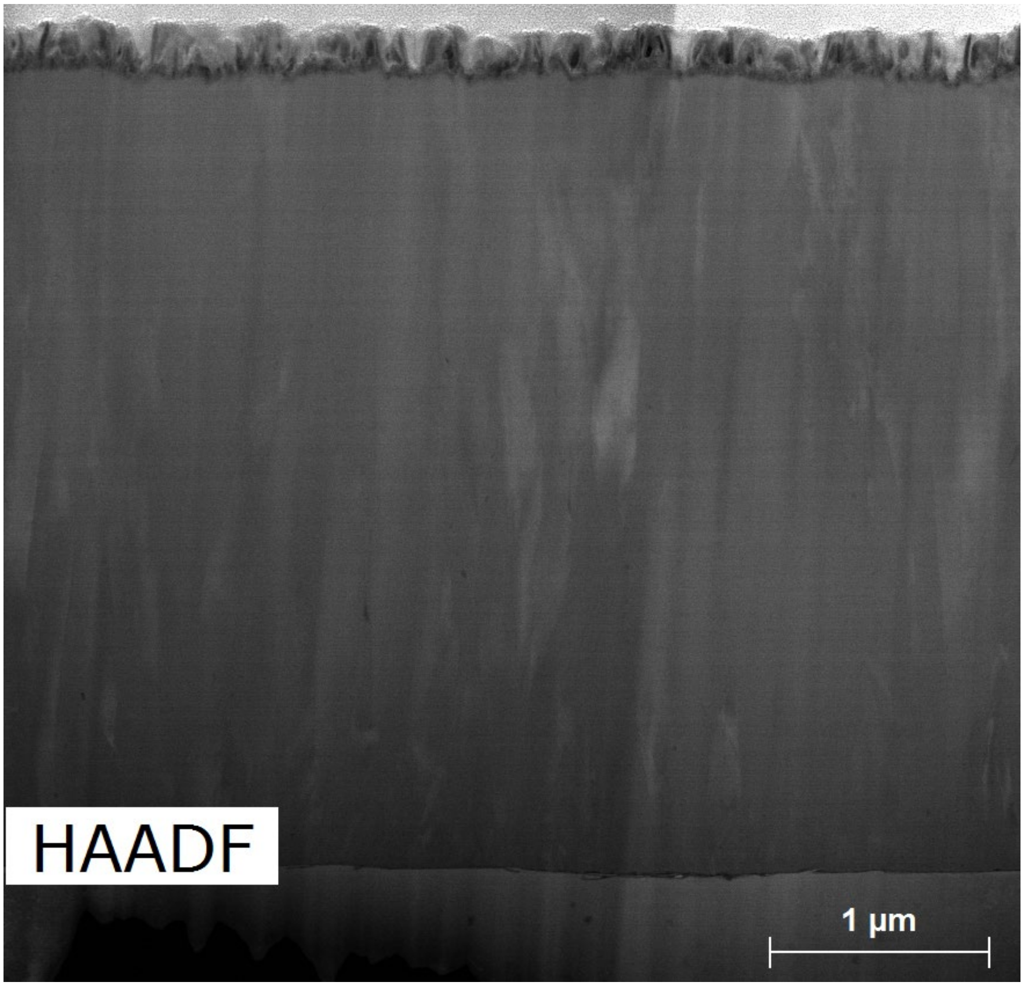

Supplementary Fig. S6: HAADF (High-Angle Annular Dark Field) STEM Image, of a Ni-Cu_2O-Cu foil sample prepared exactly as the Ni-Cu_2O-Cu mesh electrode, without any photoelectrochemical surface modification. The thickness of the Cu_2O layer is clearly visible though the Ni nanolayer is not at this scale, despite being present. The top visible layer is the Pt cap used for sample preparation.

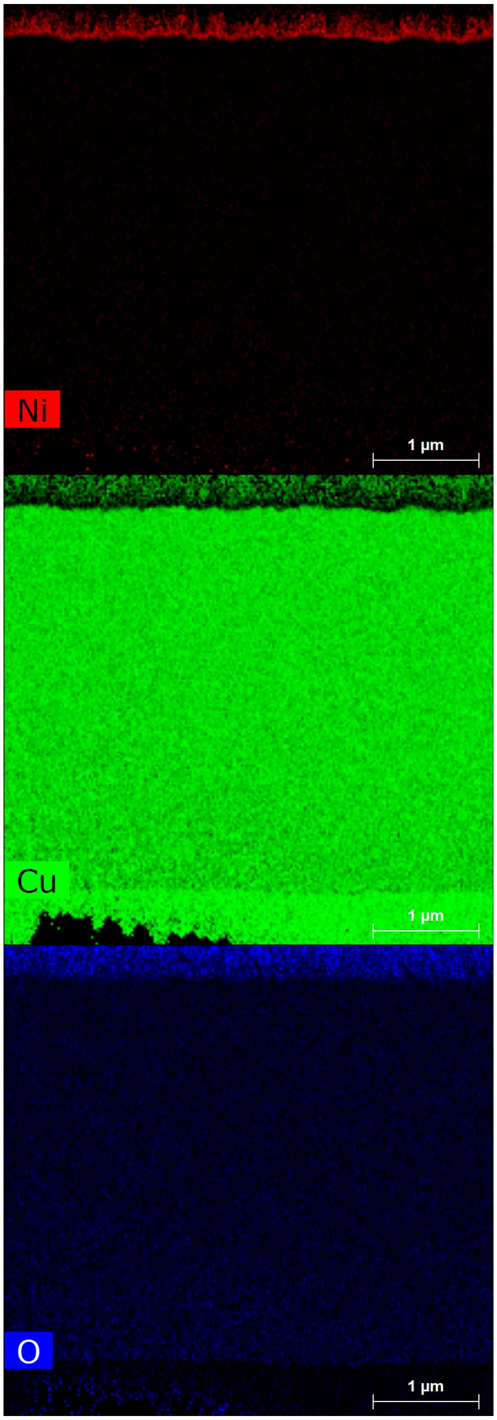

Supplementary Fig. S7: Cross section EDS maps of elemental Ni, Cu, and O in the Ni-Cu_2O-Cu foil (prepared exactly as the Ni-Cu_2O-Cu mesh electrode) without any photoelectrochemical surface modification, clearly showing the elemental composition and presence of the Ni nanolayer on top, mix of Cu and O in the Cu_2O layer, and Cu in the copper substrate. This figure also shows the approximate thicknesses of the nanostructured heterolayers.

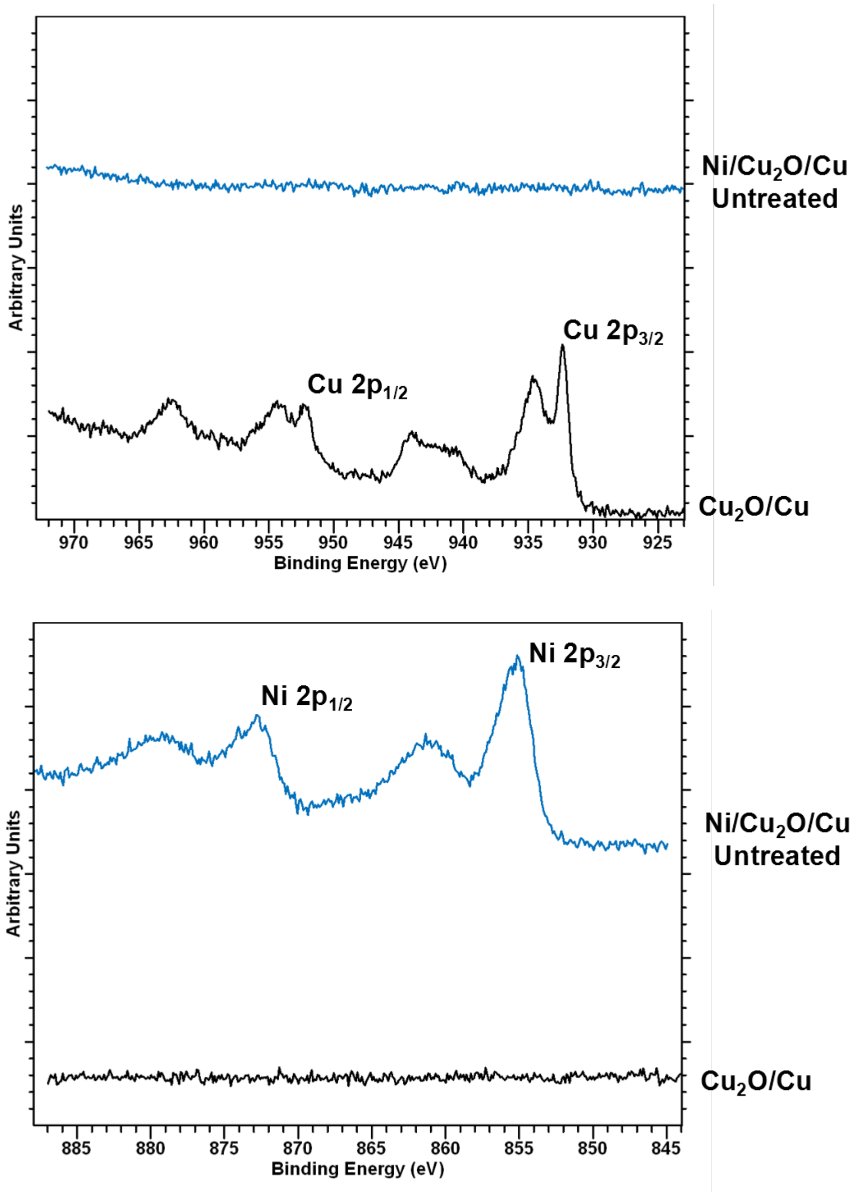

Supplementary Fig. S8: XPS spectra of Ni-Cu_2O-Cu mesh electrode <u>before</u> photoelectrochemical surface modification. The peak between 960 eV and 965 eV and structure between 940 eV and 950 eV are indicative of the presence of CuO whereas the other peaks are indicative of Cu_2O [29].

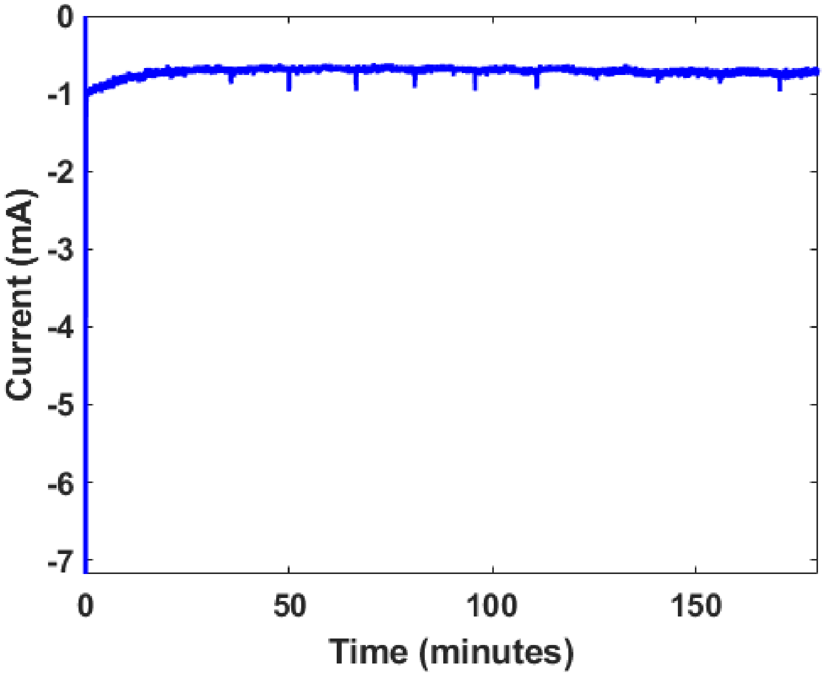

Supplementary Fig. S9: Steady state cathodic current versus time at later stages (i.e. after the time range shown in **Fig. 3b**) of the photoelectrochemical surface modification process, showing that the photoelectrochemical surface modification is complete within ~ 1 h. The ordered spikes reflect the disturbances in the current measurements that occur at each instance when 350 μL aliquots were withdrawn at regular intervals.

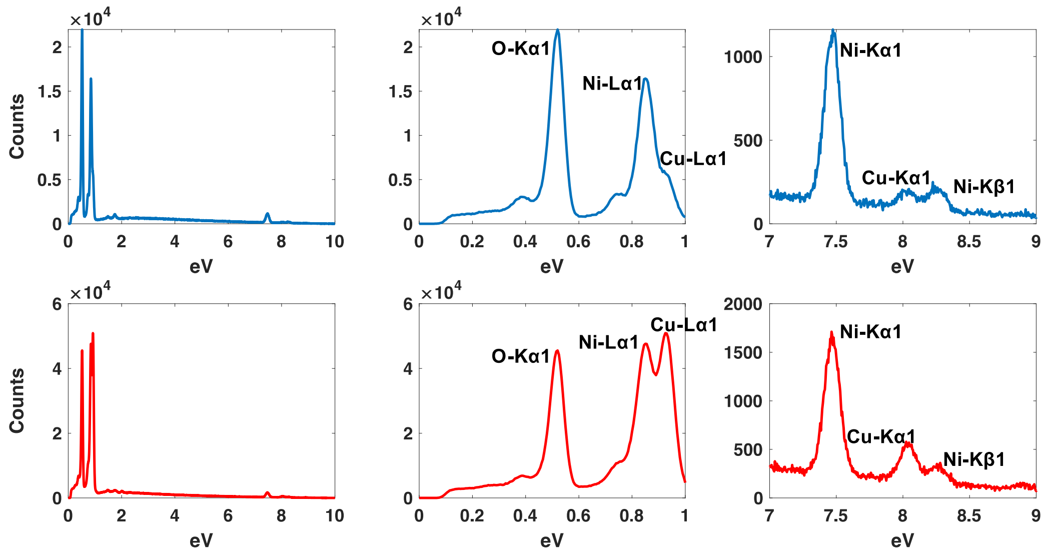

Supplementary Fig. S10: EDS spectra of the Ni-Cu_2O-Cu electrode before (top row) and after (bottom row) photoelectrochemical surface modification. Figures in the middle and right columns are expanded views of specific ranges of energies in the figures in the left column.

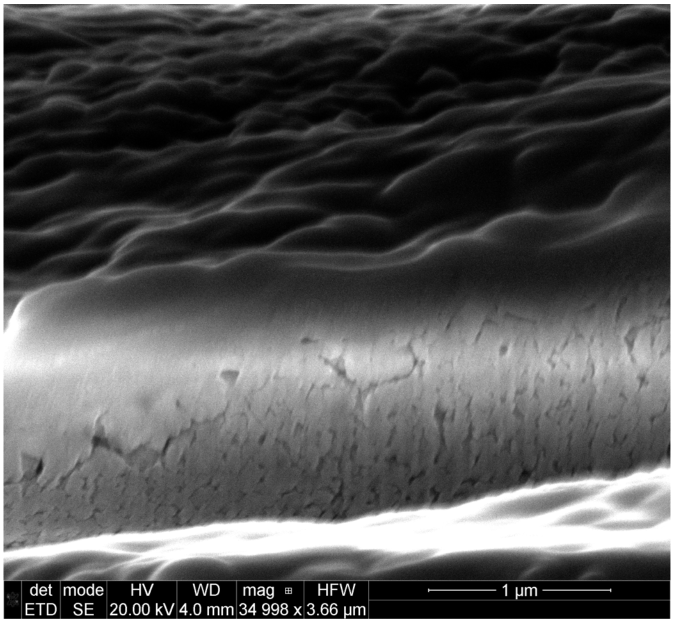

Supplementary Fig. S11: Cross-section FIB-SEM image of the Ni-Cu_2O-Cu electrode <u>after</u> photoelectrochemical surface modification

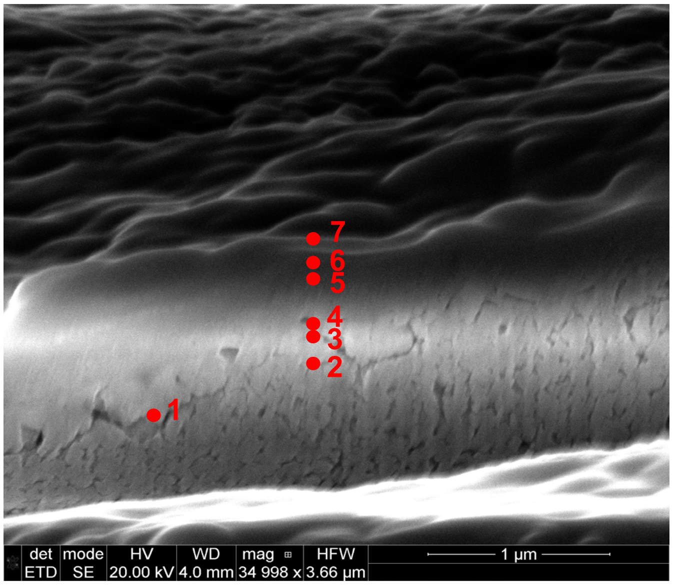

Height Location	Ni K Atomic %	O K Atomic %
**7**	6.03	-
**6**	7.64	-
**5**	10.84	7.66
**4**	3.34	-
**3**	2.44	-
**2**	1.82	-
**1**	1.24	-

Supplementary Fig. S12: Cross-section FIB-SEM image of Ni-Cu_2O-Cu electrode <u>after</u> photoelectrochemical surface modification. Point-wise EDS measurement locations are labeled in red and the corresponding elemental O and Ni atomic percentages are given in the accompanying table.

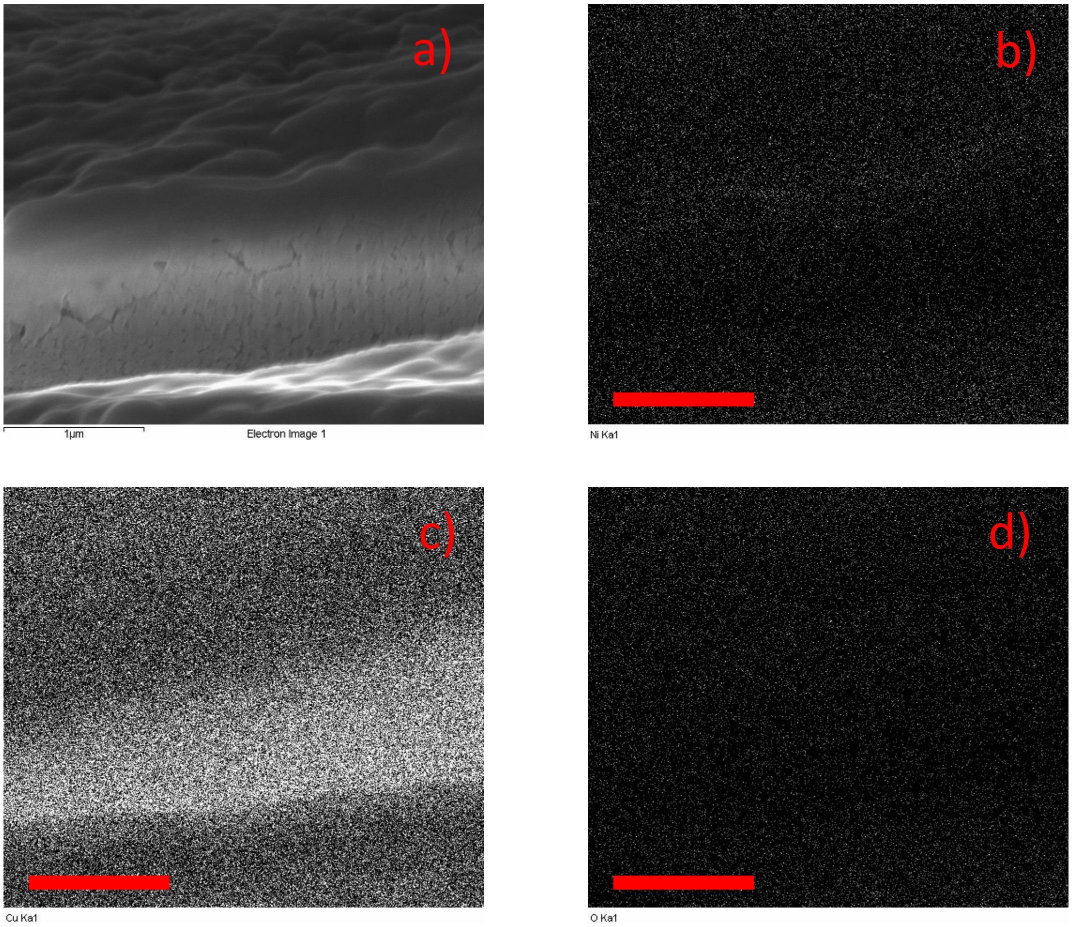

Supplementary Fig. S13: Cross-section EDS maps of elemental Ni (**top right**), Cu (**bottom left**), and O (**bottom right**) on the Ni-Cu_2O-Cu electrode after photoelectrochemical surface modification, for the sample shown in **Supplementary Fig. 11**. Scale bars are all 1 μm. Pixel intensity is indicative of greater counts for that element (i.e. darker denotes absence while lighter denotes presence).

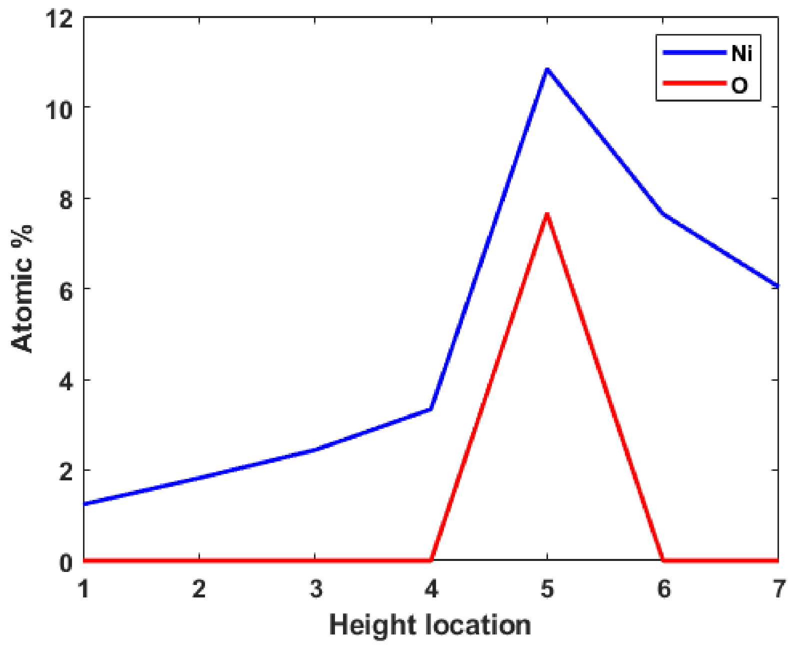

Supplementary Fig. S14: Elemental composition of Ni (blue) and O (red) obtained by EDS in the cross section of the surface layers of the Ni-Cu_2O-Cu mesh electrode after photoelectrochemical surface modification showing the oxide layer being depleted of oxygen. The locations correspond to those shown in **Supplementary Fig. 12.** An increasing height location index corresponds to a higher physical height, i.e. closer to the top surface, in **Supplementary Fig. 12.**

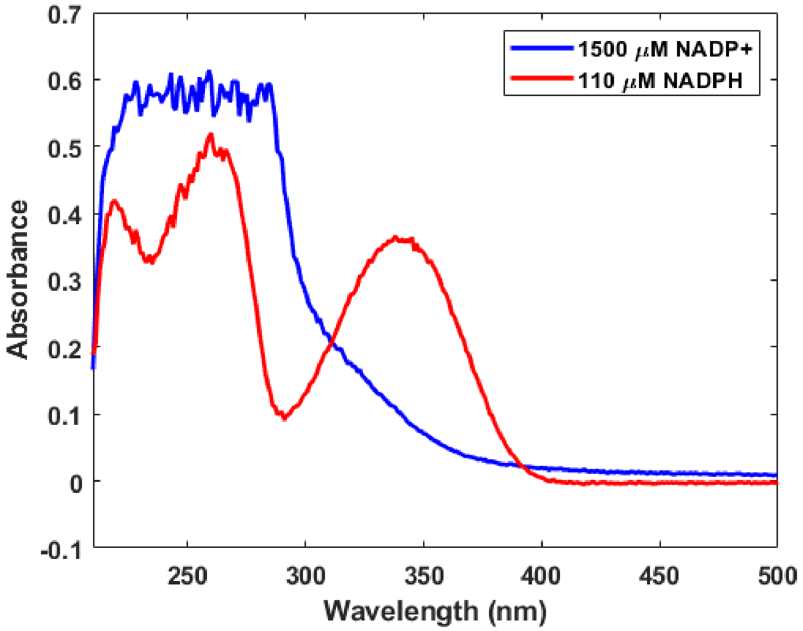

Supplementary Fig. S15: An illustrative example of an absorption spectrum used to determine the progress of cofactor regeneration using the *Lb*ADH-based selective enzymatic assay on samples aliquoted from the cathode side of the apparatus (**Fig. 3a**)

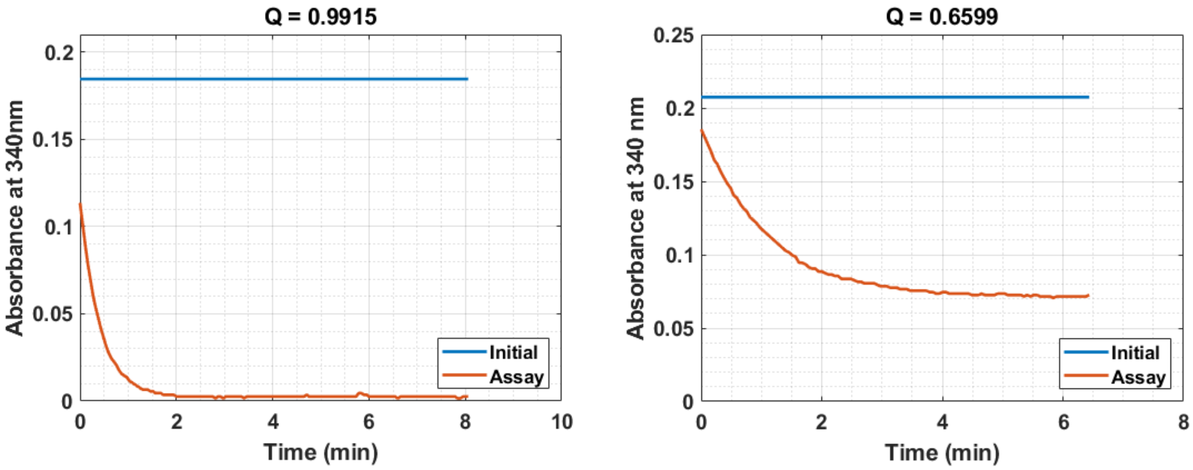

Supplementary Fig. S16: **Left:** The *Lb*ADH enzyme assay applied to a stock (pure, active) NADPH solution; **Right:** *Lb*ADH enzyme assay applied to electrochemically regenerated NADPH using an Ni-Cu_2O-Cu electrode, with an initial concentration of 1.5 mM $NADP^+$.

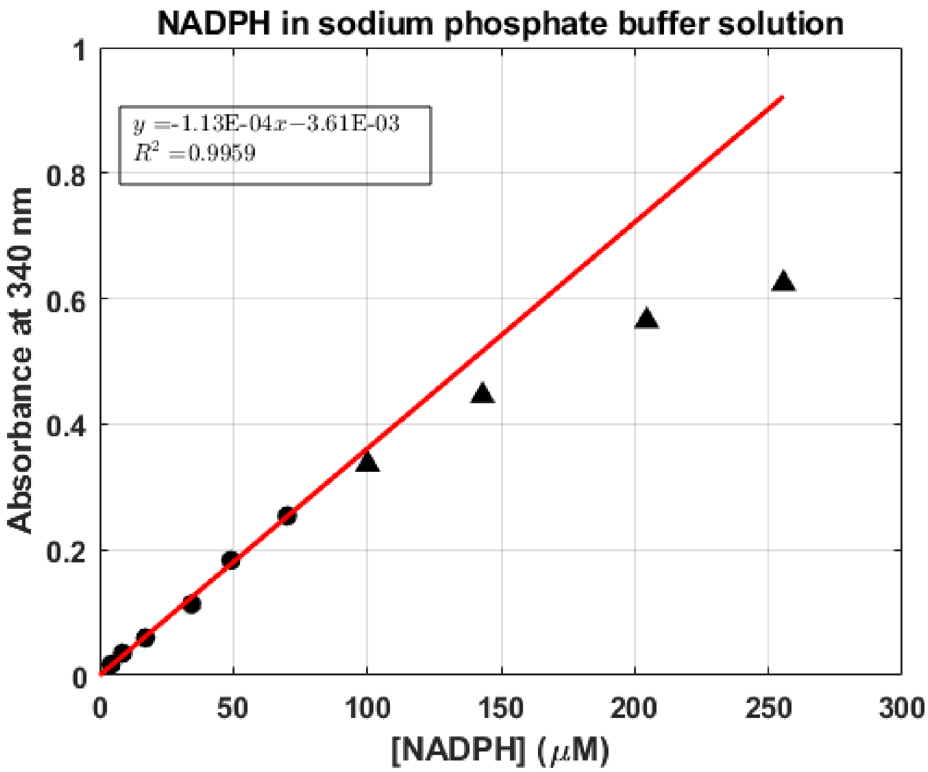

Supplementary Fig. S17: Standard NADPH absorbance-concentration calibration in sodium phosphate buffer solution (pH 8). Only points denoted with circles were fit to yield the calibration equation shown in the box.
